# Effects of plastic film mulching on the spatiotemporal distribution of soil water, temperature, and photosynthetic active radiation in a cotton field

**DOI:** 10.7717/peerj.13894

**Published:** 2022-09-01

**Authors:** Beifang Yang, Lu Feng, Xiaofei Li, Guozheng Yang, Yunzhen Ma, Yabing Li

**Affiliations:** 1 Institute of Cotton Research, Chinese Academy of Agricultural Sciences/State Key Laboratory of Cotton Biology, Anyang, Henan, The People’s Republic of China; 2 College of Plant Science and Technology, Huazhong Agricultural University, Wuhan, Hubei, The People’s Republic of China; 3 Zhengzhou Research Base, State Key Laboratory of Cotton Biology, School of Agricultural Sciences, Zhengzhou University, Zhengzhou, Henan, The People’s Republic of China

**Keywords:** Soil water content, Plastic film mulching, Soil temperature, Temporal and spatial changes, Sensors, PAR

## Abstract

Plastic film mulching (PFM) affects the spatiotemporal distribution of soil moisture and temperature, which in turn affects cotton growth and the spatiotemporal distribution of canopy photosynthetically active radiation (PAR). Due to the spatial heterogeneity of soil moisture, temperature and limited monitoring methods, the issues such as relatively few sampling points and long sampling intervals in most existing studies prevent the accurate quantification of spatiotemporal changes in moisture and temperature along soil profile. To investigate the effects of PFM on spatiotemporal changes in soil moisture, temperature, and canopy PAR in cotton fields, two field trials of plastic film-mulched (M) and nonmulched (NM) cultivations were performed in 2018 and 2019. The grid method was used for the soil information continuous monitoring and multiple-time fixed-site canopy PAR monitoring during the duration of cotton growth. Two-year field trial data showed that, M cultivation increased soil moisture by approximately 13.6%–25% and increased temperature by 2–4 °C in the 0–50 cm soil layer before the first irrigation (June 20) and by 1–2 °C in the 70–110 cm soil layer, compared with NM cultivation. In addition, the temperature difference between the two treatments gradually decreased with the increase in irrigation and air temperature. The M treatment reached the peak PAR interception rate 10 days earlier than the NM treatment. In 2018 and 2019, the PAR peak value under the M treatment was 4.62% and 1.8% higher than that under the NM treatment, respectively, but the PAR interception rate was decreased rapidly in the late growth stage. Overall, PFM had an effect on soil moisture retention during the whole growth period and greatly increased the soil temperature before budding stage, thus promoted the early growth of cotton. Considering this, we suggest that the irrigation quota and frequency could be appropriately decreased in the case of plastic film mulching cultivation. For nonmulching cultivation, the irrigation quota and frequency should be increased, and it is necessary to take measures to improve the soil temperature before middle July.

## Introduction

Cotton is an economically important crop in China. China’s total cotton production accounts for approximately 24.8% of the world’s cotton yield (Mao & Li, 2016). Xinjiang is a main cotton-producing area in China; the cotton planting area and output in Xinjiang accounts for approximately 76% and 83.7% of that in China, respectively (NBS.National Bureau of Statistics, 2019).

Soil moisture and temperature affect the crop growth, and it is very important to study the spatiotemporal distribution of soil moisture and temperature in different planting patterns to improve cotton production potential and water use efficiency (Xing et al., 2020a; Xing et al., 2020b; Venugopalan et al., 2010). Previous studies have shown that the distribution of soil water and temperature exhibit spatial heterogeneity (Liu et al., 2020) and spatial correlation (Wang et al., 2009). With increasing soil depth, the spatial correlation of the soil water content decreased, and the soil moisture varied with the distances from the cotton row and soil depths (Li et al., 2010). Plastic film mulching (PFM) is known as one of the revolutionary technologies of agricultural production, and it is also an important technical measure for cotton production in the Xinjiang cotton area to obtain high yield and quality (Dai & Dong, 2014; Chen et al., 2019). Studies have shown multiple advantages of PFM, such as reducing soil water evaporation (Gao & Li, 2005) and increasing topsoil moisture by 23. 83% (Zong et al., 2021), redistributing soil moisture to alleviate drought stress (Li et al., 2004; Yan & Li, 2016), increasing topsoil temperature (Wang, Kang & Liu, 2003; Ghosh et al., 2006), especially in the early and middle stages of cotton growth (Zong et al., 2020), improving root zone salinity status (Dong et al., 2008) and raising the seedling rate (Feng et al., 2015). PFM has been widely used in the production of wheat (Zhang et al., 2019), corn (Lee et al., 2019; Lamptey et al., 2020), cotton (Wang et al., 2019) and other crops, especially in rain-fed agricultural areas or arid/semiarid areas (Dong et al., 2009).

However, there are some problems such as relatively few sampling points and long sampling intervals in most existing studies on the spatiotemporal distribution of soil moisture. For example, Wang et al. (2009) monitored the soil water content only seven times during the whole growth period to investigate the distribution of surface soil moisture and irrigation uniformity. Using geostatistical methods, Shen et al. (2010) studied the spatial variation of soil moisture in drip irrigation conditions under the plastic film of the plastic film-mulched cotton field by dividing 1 meter-depth soil into 5 layers and sampling only once. Wang et al. (2015) and Hu et al. (2020a); Hu et al. (2020b) examined the spatial variation and seasonal changes in soil moisture by sampling only once for each growth period. The relatively few sampling times in these studies prohibit them from continuously monitoring the spatiotemporal variation trend of soil moisture.

In addition, most of the existing studies only focus on the spatial distribution of soil moisture, and there are few studies on soil temperature and canopy photosynthetically active radiation (PAR). There are even fewer studies on soil moisture, temperature, and canopy PAR by field experiments of film mulching and nonmulching cotton cultivation. Given this, we conducted continuous grid-based monitoring of soil water content, temperature, and canopy PAR under plastic film-mulched (M) treatment and nonmulched (NM) treatment during the whole growth period. The purpose of this study was to reveal the spatiotemporal effects of PFM on soil moisture, temperature, and canopy PAR in cotton fields. Our results provided a theoretical basis for cotton production and the precise management of water and fertilizer in the Xinjiang cotton area. Results will contribute to the improvement of water and fertilizer utilization efficiency, thus promoting the sustainable development of agriculture in this area.

## Material and Methods

### Field trial site

The field trials were conducted at the Alar Comprehensive Test Station (40°36′N, 81°19′E) of the Institute of Cotton Research of Chinese Academy of Agricultural Sciences, the First Division of the Xinjiang Production and Construction Corps from 2018 to 2019. This area is located at the southern foot of the Tianshan Mountains and the northern part of the Tarim Basin. It belongs to a warm temperate continental arid desert climate with an average annual temperature of 10.7 °C, solar radiation of 5.59 × 10^9^–6.12 × 10^9^ J/m^2^, sunshine of 2,556.3–2,991.8 h, precipitation of 40.1–82.5 mm, and annual average evaporation of 1,876.6–2,558.9 mm (PGAM, 2022). Soil texture of the site is mainly sandy loam soil. The 0–20 cm soil contained organic matter (10.58 g/kg), alkalihydrolyzable nitrogen (84.87 mg/kg), total nitrogen (0.64 g/kg), available phosphorus (25.38 mg/kg), and available potassium (190.5 mg/kg) with a pH of 7.7. The total rainfall and sunshine duration during cotton growing seasons (April–October) was 71.5 mm, 1,803.5 h and 93 mm, 1,716.7 h in 2018 and 2019, respectively (NMC, 2021).

### Experimental design

In order to study the effects of plastic film-mulched (M) treatment on soil moisture, temperature, and canopy PAR in cotton fields, field trials were conducted with the nonmulched (NM) treatment as a control, each treatment was repeated three times. CCRI-92 (from Institute of Cotton Research of Chinese Academy of Agricultural Sciences) was used as the test variety. The sowing date was April 16, 2018 and April 19, 2019, respectively. Each test plot contained five films with one film covering six rows in east–west direction. The planting pattern was as follows: the row spacing was 66 cm+10 cm; the row length was 7 m; the area was 79.8 m^2^; and the density was 180,000 plants ha^−1^. The plastic film used in this study was white transparent polyethylene film with a width of 2.05 m, and a thickness of 0.01 ± 0.002 mm and the transmittance and thermal emissivity of film were more than 90%.

### Data collection

**Figure 1:**
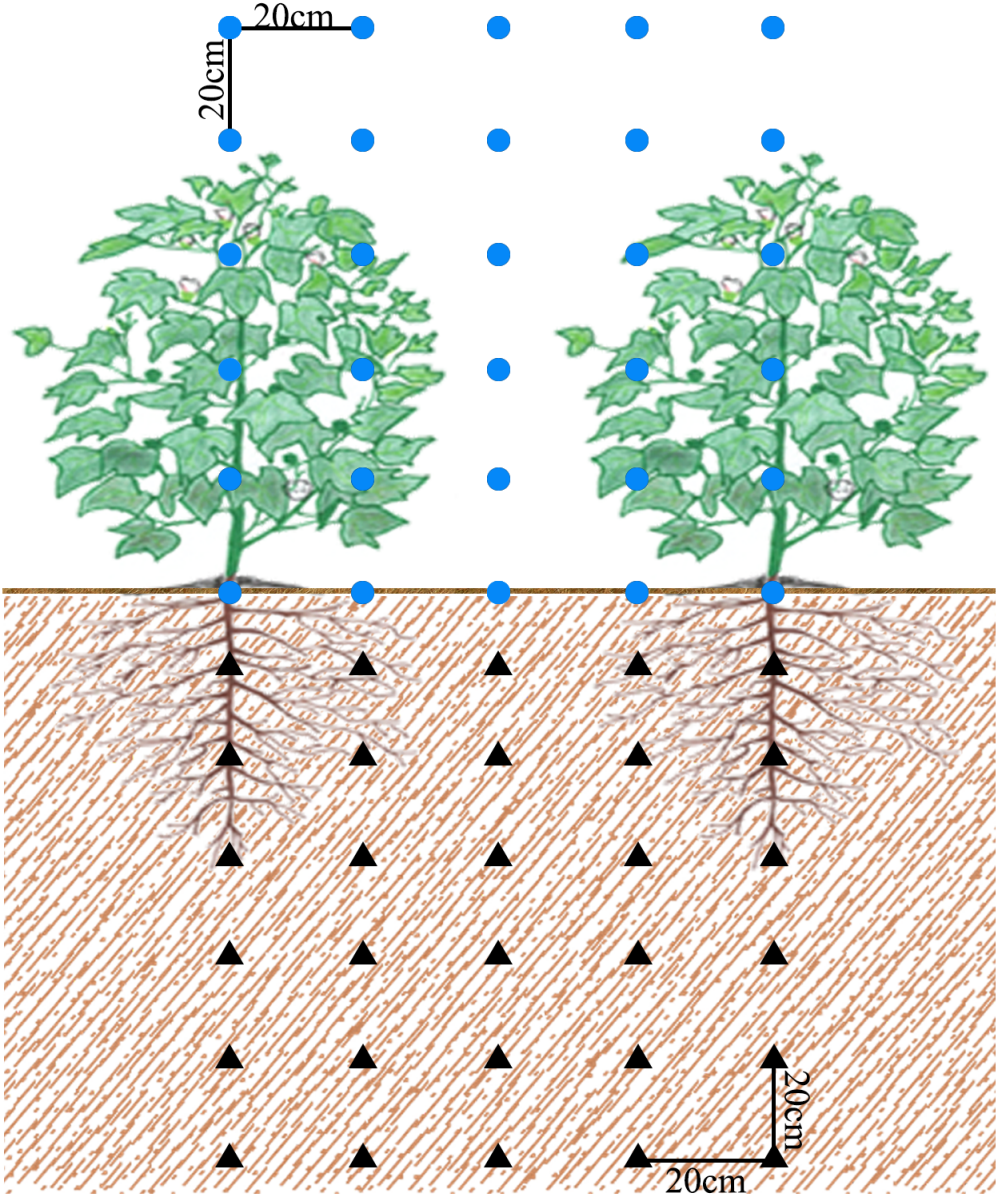
Schematic diagram of monitoring points of field soil and canopy PAR. Blue circle dots and black triangles represent canopy PAR monitoring points and soil moisture, temperature monitoring points, respectively.

The soil information of soil moisture, temperature, and conductivity was acquired by three-parameter sensors 5TE (Decagon Devices, Inc., Hopkins, NE, USA) and programmable data collectors CR1000 (Campbell Scientific, Inc., Logan, UT, USA). According to the spatial grid method, sensors were arranged in the soil section perpendicular to the direction of cotton rows (the black triangles in Fig. 1). A total of 30 sensors were arranged with one sensor every 20 cm in the horizontal direction at each layer. There were six layers in the vertical direction with depths of 10 cm, 30 cm, 50 cm, 70 cm, 90 cm, and 110 cm. Data were recorded every hour. Solar power supply was adapted for long-term continuous monitoring. Unfortunately, due to the failure of the solar power supply system during the monitoring period, the NM data were missing from July 8 to August 1 in 2018.

The 100 cm long rod-shaped PAR sensors LI-191SA (LI-COR, Lincoln, NE, USA) and LI-1500 data collectors LI-1500 (LI-COR, Lincoln, NE, USA) were used to measure the canopy PAR distribution from 12:00 to 14:00 under clear and windless weather. The measurement was conducted from the incident light and reflected light of each monitoring point, point by point according to the blue circle dots in Fig. 1 from bottom right to top left. The PAR sensors were kept parallel to the cotton planting row, and the photosensitive surface of the sensor was constantly kept in the horizontal direction. The measurement was conducted once every 15 days.

### Data processing

In this study, soil moisture, temperature, and canopy PAR data were first preprocessed by Stata software (Stata Corp LP, College Station, Texas, USA). Since the data of soil water content was calculated by the dielectric permittivity (*ɛ*_a_) measured by 5TE (5TE, Decagon Devices, Inc., Hopkins, NE, USA). Normally, the sensor needs to be calibrated in advance by a manual on-site sampling method if the soil conductivity to be measured is high (>10 ds/m); in other cases, the soil water content can be calculated by Topp equation (Eq. (1)) (Topp, Davis & Annan, 1980). In addition, the data of the whole growth period were recorded in one document, and the amount of data is large. According to our sensor arrangement, 30 data were divided into a group. To obtain each group of data by hour, the original data was converted according to Eq. (1) and separated them by different time intervals. Finally, a series of single group data were obtained and saved as a ‘.dat’ format file for subsequent interpolation processing.

(1) \begin{eqnarray*}\mathrm{V WC}=4.3\times 1{0}^{-6}{}_{\mathrm{ a}}^{3}-5.5\times 1{0}^{-4}{}_{\mathrm{ a}}^{2}+2.92\times 1{0}^{-2}{}_{\mathrm{ a}}-5.3\times 1{0}^{-2}\end{eqnarray*}

where VWC is the volumetric water content of the point to be measured and *ɛ*_a_ is the dielectric permittivity measured directly by the sensor.

To obtain the spatial distribution of soil moisture, temperature, and canopy PAR interception rate, the unmeasured points needed to be interpolated according to measured points. This study used the Kriging interpolation method in Surfer13 (Golden Software Inc., Golden, CO, USA) software to estimate the value of the unmeasured points by Eq. (2) (Li et al., 2012):

(2) \begin{eqnarray*}\sum _{\mathrm{i}=1}^{\mathrm{n}}{\lambda }_{\mathrm{ i}}\gamma \left( {\mathrm{X}}_{\mathrm{i}},{\mathrm{X}}_{\mathrm{j}} \right) +\varnothing =\gamma \left( {\mathrm{X}}_{\mathrm{i}},{\mathrm{X}}_{0} \right) i=1,2,\ldots ,n\end{eqnarray*}

where $\gamma \left( {\mathrm{X}}_{\mathrm{i}},{\mathrm{X}}_{\mathrm{j}} \right) $ is the semivariation value between observation points X_i_ and X_j_; *φ* isthe Lagrange operator; $\gamma \left( {\mathrm{X}}_{\mathrm{i}},{\mathrm{X}}_{0} \right) $ is the variation function value between the known point and the unknown point; $\gamma \left( {\mathrm{X}}_{\mathrm{i}},{\mathrm{X}}_{\mathrm{j}} \right) $ is the value of the variation function between the known points, and *X*_0_ isthe estimated value of the point to be measured.

The PAR interception rate (iPAR), transmittance (tPAR), and reflectance (rPAR) of each layer of cotton canopy were calculated using the method of Tang et al. (2012), who divided the input PAR from the top of the canopy into three parts: one part was reflected by canopy; the second part was transmitted from the canopy to the ground; and the third part was intercepted by the canopy. Therefore, the following equation was used to calculate iPAR:

(3) \begin{eqnarray*}iPAR=1-tPAR-rPAR.\end{eqnarray*}

The cotton population canopy PAR interception rate and soil moisture content in the monitoring area were calculated using the 3/8 extended Simpson rule in Surfer13 software (Li et al., 2012; Wang et al., 2021a; Wang et al., 2021b). The equation is as follows:

(4) \begin{eqnarray*}{\mathrm{A}}_{\mathrm{i}}& = \frac{3\Delta \mathrm{X}}{8} \left( {\mathrm{G}}_{\mathrm{i},1}+3{\mathrm{G}}_{\mathrm{i},2}+\mathrm{G}{3}_{\mathrm{i},3}+2{\mathrm{G}}_{\mathrm{i},4}+\cdots +2{\mathrm{G}}_{\mathrm{i},\text{ncol}-1}+{\mathrm{G}}_{\mathrm{i},\text{ncol}} \right) \end{eqnarray*}

(5) \begin{eqnarray*}\mathrm{V }& \approx \frac{3\Delta \mathrm{y}}{8} \left( {\mathrm{A}}_{1}+3{\mathrm{A}}_{2}+3{\mathrm{A}}_{3}+2{\mathrm{A}}_{4}+\cdots +2{\mathrm{A}}_{\text{ncol}-1}+{\mathrm{A}}_{\text{ncol}} \right) \end{eqnarray*}

where Gij is the quantized value at the i-th row and j-th column of the monitor profile; A_i_ is the i-th cross-sectional area; ΔX and Δy are the column spacing and row spacing of the grid data file, respectively; and V is the total amount of PAR or water content on the monitored soil profile. The coefficients of Eqs. (4) and (5) are [1, 3, 3, 2, ……3, 3, 2, 1].

### Statistical analysis

The analysis of variance (ANOVA) of temperature and temperature differences in different growth stages and canopy PAR interception rates was performed using SPSS 16.0 (SPSS Inc., Chicago, IL, USA). Differences between treatments were considered significant at *P* < 0.01 and *P* < 0.05 according to least significant differences (LSD).

## Results

### Spatiotemporal distribution of soil moisture

#### Change trend of soil moisture over time

**Figure 2:**
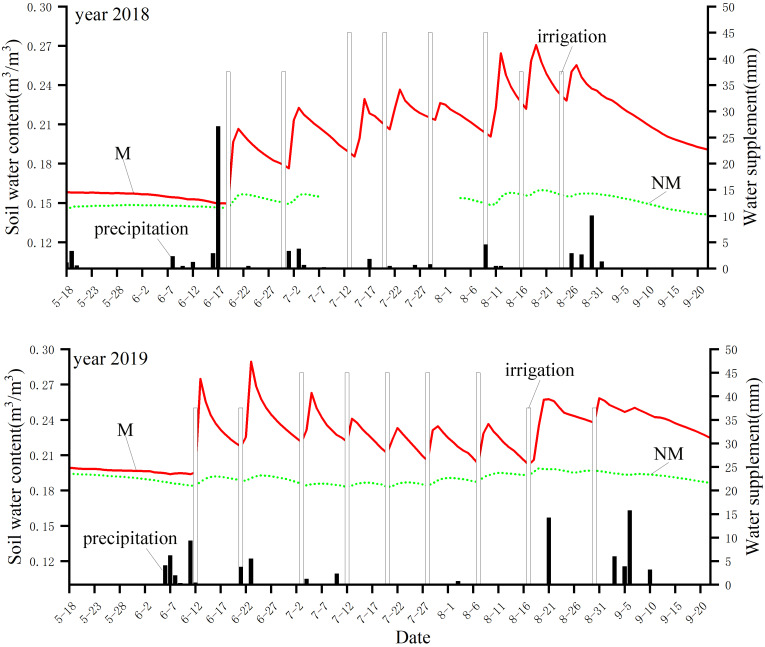
Curve of soil moisture change and water supplement with time in 2018 and 2019.

To clearly show the change in soil moisture under the two treatments in 2018 and 2019 over time, we represented the soil water content over the whole growth period with a curve, and the irrigation and precipitation information is represented by a histogram (Fig. 2). Figure 2 shows that the soil moisture under the M treatment in 2018 exhibited a trend of decline before the first irrigation, volatility rise in the middle stage and steady decline in the later stage. In 2019, it showed a trend of decline before the first irrigation, fluctuation in the middle stage and decline in the later stage. The trend of the NM treatment was consistent in two years, both of which showed a trend of slow decline before the first irrigation, fluctuation in the middle stage and steady decline in the later stage. In addition, the M treatment showed obvious changes with irrigation and water consumption; that is, the soil moisture increased rapidly after each irrigation and then gradually decreased with consumption, while the NM treatment exhibited little change in soil moisture during the whole cotton growth period, and soil moisture varied slightly with irrigation and water consumption.

Specifically, in 2018, the soil water content under the M treatment fluctuated at approximately 0.2 m^3^/m^3^ while under the NM treatment, the soil moisture fluctuated at approximately 0.15 m^3^/m^3^, which was approximately 25% lower than that under the M treatment. In 2019, the soil moisture under both the M treatment and NM treatment was higher than that in 2018, and the soil moisture under the M treatment fluctuated approximately 0.22 m^3^/m^3^, which was approximately 10.0% higher than that in 2018, while the soil moisture under the NM treatment fluctuated approximately 0.19 m^3^/m^3^, which was approximately 26.7% higher than that in 2018. The soil moisture under NM treatment was approximately 13.6% lower than that under the M treatment in 2019.

#### Change trend of soil moisture and its difference with time in different soil layers

**Figure 3:**
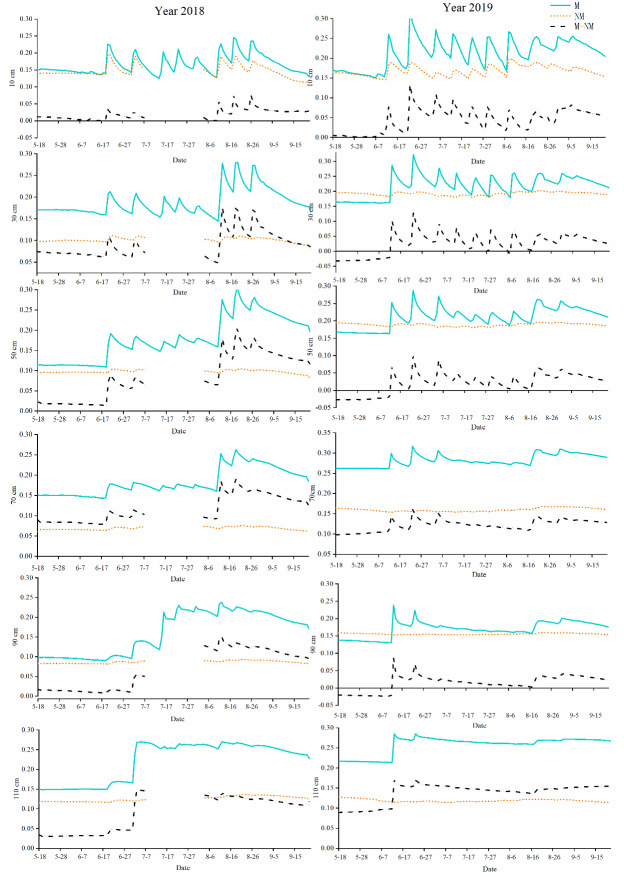
Curve of soil moisture(m^3^/m^3^) and moisture difference change over time in different depths in 2018 and 2019.

PFM affected the distribution of soil moisture in the vertical direction. The changes in soil moisture at different soil depths with time and the distribution in different growth stages are shown in Fig. 3 and Table 1. As shown in Fig. 3, PFM significantly increased the soil water content in the cotton field after the first irrigation (June 20th), and then with irrigation and water consumption, the soil water content in the 0–90 cm soil layer changed significantly, and the soil water content exhibited the most significant change in the 0–50 cm soil layer, ranging from 0.15−0.3 m^3^/m^3^. Soil moisture content in the nonmulched cotton field was basically unchanged in all the soil layers except the 0–30 cm soil layer. PFM mainly increased the soil water content by 0.1−0.2 m^3^/m^3^ in the soil layers above 70 cm in the short term. There were significant differences in the soil water content and water content between the different treatments in the same growth period (Table 1). The minimum water content difference between treatments was approximately 0−0.08 m^3^/m^3^ in the seedling stage. With cotton growth, the difference in water content displayed an increasing trend and reached a maximum in the boll opening stage, which was approximately 0.01−0.15 m^3^/m^3^.

### Spatial distribution of soil moisture in different growth stages

**Figure 4:**
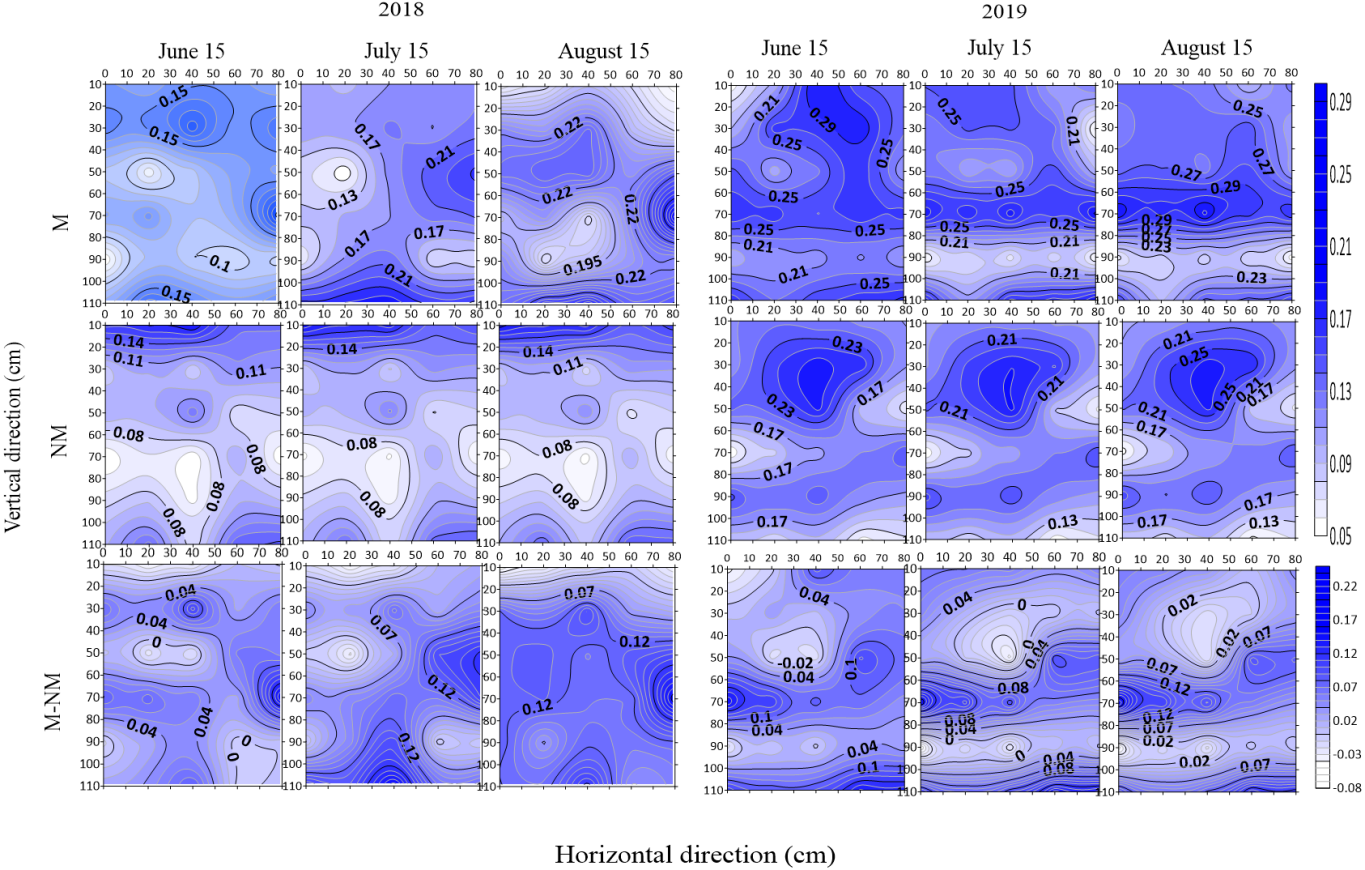
Spatial distribution of soil moisture(m^3^/m^3^) and moisture difference in different growth stages in 2018 and 2019.

The spatial distribution of soil moisture under the two treatments in different growth stages (June 15, budding stage; July 15, full blooming stage; August 15, full bolling stage) in 2018 and 2019 is shown in Fig. 4. Overall, there were differences in the spatial distribution of soil moisture in the monitored soil profile. In 2019, the soil moisture in each monitored soil profile was higher than that in 2018. The soil moisture under the M treatment in each year was higher than that under the NM treatment. The top-layer soil moisture was higher than the deep-layer moisture, and longitudinal change in the moisture under different treatments showed a high-low-high trend. The soil moisture at the 0–30 cm soil layer changed drastically in different growth stages, and that at other layers showed different change trends under different treatments.

**Table 1:** Soil moisture and moisture difference in different growth stages in 2018 and 2019.

**Year**	**Soil depth** (cm)	Seeding stage (m^3^/m^3^)			Budding stage (m^3^/m^3^)			Flowering and Boll -forming stage (m^**3**^/m^**3**^)			**Boll opening stage (m^**3**^/m^**3**^)**		
		**M**	**NM**	**TD**	**M**	**NM**	**TD**	**M**	**NM**	**TD**	**M**	**NM**	**TD**
**2018**	0-20	0.149d	0.098c	0.051c	0.156b	0.099c	0.057c	0.26a	0.11b	0.15a	0.246a	0.111b	0.135b
	20–40	0.098f	0.083e	0.015e	0.096f	0.084e	0.012e	0.196d	0.09e	0.106c	0.194e	0.087e	0.107d
	40–60	0.15c	0.066f	0.084a	0.156c	0.067f	0.089a	0.194e	0.072f	0.121b	0.211c	0.067f	0.144a
	60–80	0.114e	0.095d	0.018d	0.133e	0.098d	0.035d	0.2b	0.1d	0.099d	0.228b	0.094d	0.134c
	80–100	0.171a	0.099b	0.072b	0.173a	0.102b	0.071b	0.196c	0.105c	0.092e	0.197d	0.096c	0.101e
	100–120	0.151b	0.16a	−0.01f	0.156d	0.167a	−0.011f	0.177f	0.18a	−0.004f	0.16f	0.151a	0.009f
**2019**	0–20	0.167c	0.162d	0.004c	0.201d	0.166c	0.035c	0.222d	0.171c	0.051c	0.234d	0.168c	0.066c
	20–40	0.164e	0.225a	−0.061f	0.215c	0.222a	−0.007f	0.225c	0.221a	0.004f	0.236c	0.224a	0.012f
	40–60	0.167d	0.193b	−0.027e	0.2e	0.189b	0.012e	0.218e	0.187b	0.031d	0.231e	0.19b	0.041d
	60–80	0.262a	0.163c	0.099a	0.276a	0.157d	0.119b	0.284a	0.159d	0.125b	0.298a	0.165d	0.133b
	80–100	0.137f	0.158e	−0.021d	0.168f	0.155e	0.013d	0.172f	0.155e	0.017e	0.189f	0.157e	0.032e
	100–120	0.216b	0.126f	0.09b	0.251b	0.117f	0.134a	0.265b	0.118f	0.147a	0.27b	0.117f	0.153a

**Notes.**

Different lowercase letters indicate significant differences at *P* < 0.05 level between different soil layers in each year.

TD represents soil moisture difference between M treatment and NM treatment.

Soil water content in the film mulched cotton field was higher than that in the nonmulched cotton field during the entire growth period. With cotton growth, the soil water content difference increased from 0.05 m^3^/m^3^ in the budding stage to 0.1 m^3^/m^3^ in the full bolling stage. The soil water content in the 0–50 cm soil layer increased by 0.05 m^3^/m^3^, while that in the soil layer below 50 cm increased by approximately 0.08−0.15 m^3^/m^3^. These results indicated that in the long run, film mulching mainly increased the soil water content in the middle and lower soil layers.

### Temporal and spatial distribution of soil temperature

#### Change trend of temperature and temperature difference with time in different soil layers

**Figure 5:**
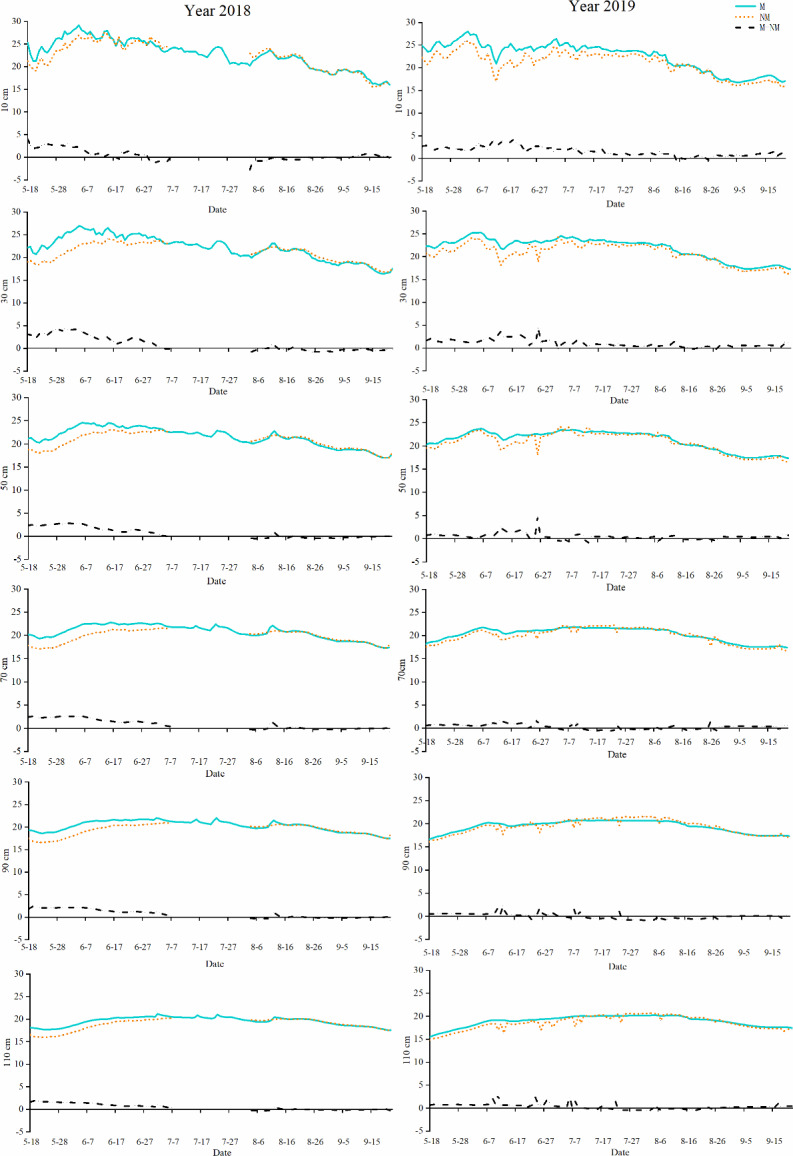
Curve of soil temperature (°C) and temperature difference change over time in different depths in 2018 and 2019.

The temperature and the temperature difference in different soil layers between treatments in 2018 and 2019 were shown in Fig. 5 and Table 2. Overall, the temperature in each soil layer under the different treatments in 2018 was higher than that in 2019. The temperature of each soil layer reached the highest value in the budding stage (Fig. 5) and then decreased gradually with the growth progression. Under each treatment, the temperature of the upper layer soil rose rapidly. In most cases, the temperature of the upper layer soil was higher than that of the lower soil layer. The temperature of each soil layer under the M treatment rose faster than that under the NM treatment, and the maximum temperature of each soil layer under the M treatment was higher than that of the corresponding soil layer under the NM treatment. Under the M and NM treatments, the upper soil reached the highest temperature in the budding stage, while the lower layer soil reached the highest temperature later than the upper soil in the flowering and boll-forming stages. Under both treatments (M and NM), the temperature showed a downward trend with increasing of soil depth. Both treatments exhibited a greater temperature difference in the seedling and budding stages than in the flowering and boll-forming stage and boll opening stage and a greater temperature difference in the upper soil than in the lower soil.

Specifically, before the boll opening period, the soil temperature gradually decreased with the increasing soil depth under each treatment, but the rate of temperature decrease was obviously different. As shown in Table 2, for both treatments, the temperature in the 0–60 cm soil layer was decreased by 1.5−3 °C with every 20 cm decrease in soil depth. The temperature in the 60–120 cm soil layer decreased by approximately 1−1.5 °C with every 20 cm decrease in soil depth. However, the soil temperature increased slightly with increasing soil depth during the boll opening stage. The rate of temperature increase varied with the soil depth, and the highest temperature of each soil layer was different. The temperature differences during different growth periods at different soil depths were also different between the M treatment and NM treatment. Temporally, greater temperature differences (0.5−3.5 °C) were detected in seedling stage and budding stage compared with the other two stages (0−1 °C). Spatially, the temperature difference decreased with increasing soil depth. The 0–40 cm soil layer displayed a relatively large temperature difference (1.6−3.5 °C) between the two years, but the 40–120 cm soil layer exhibited a relatively small temperature difference (0.5−2.5 °C).

### Spatial distribution of soil temperature in different growth stages

**Figure 6:**
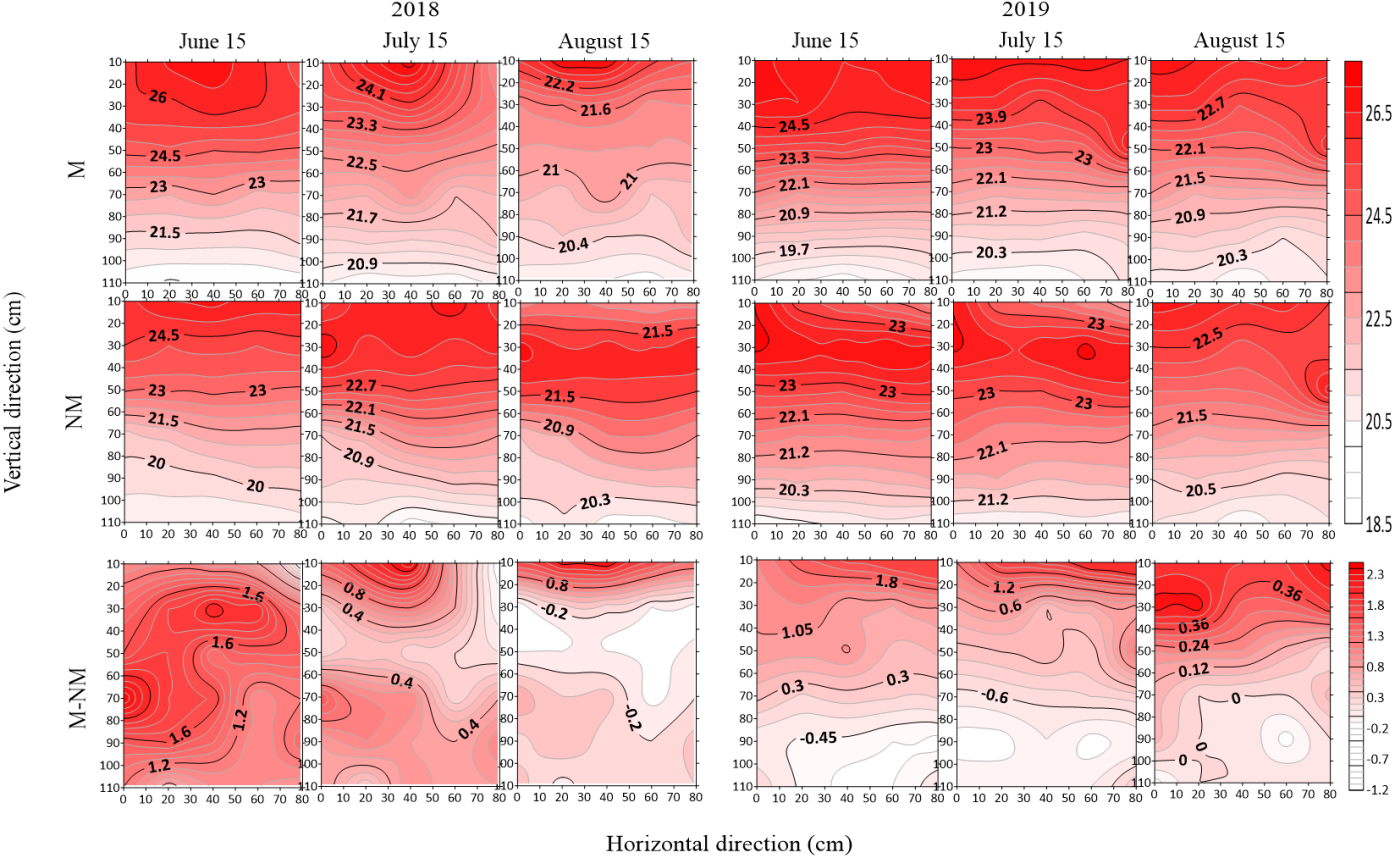
Spatial distribution of soil temperature (°C) and temperature difference in different growth stages in 2018 and 2019.

The spatial distribution of the soil profile temperature and temperature difference in two years (2018–2019) under two different treatments in different growth stages (June 15, budding stage; July 15, full blooming stage; and August 15 full bolling stage) is shown in Fig. 6. In vertical direction, the soil temperature showed a gradual decline from the surface layer to the deep layer under different treatments in different stages, and the temperature decreased by 3−5 °C under the M treatment and by 2.5−3 °C under the NM treatment. In addition, the contour line of the 0–30 cm surface soil was irregular, indicating a dramatic change in soil temperature at this soil layer. In the horizontal direction, the temperature at different soil layers was relatively stable under both the M and NM treatments in different periods except in the 0–30 cm soil layer, which exhibited a relatively large temperature change. With cotton growth progression, the temperature difference between the upper and lower soil layers gradually decreased.

**Table 2:** Soil temperature and temperature difference in different growth stages in 2018 and 2019.

**Year**	**Soil depth** **(cm)**	**Seeding stage (**° C**)**			**Budding stage (**° C**)**			**Flowering and Boll- forming stage (**° C**)**			**Boll opening stage (**° C**)**		
		**M**	**NM**	**TD**	**M**	**NM**	**TD**	**M**	**NM**	**TD**	**M**	**NM**	**TD**
**2018**	0-20	24.27a	21.42a	2.86b	26.58a	23.36a	3.23a	22.21a	21.86a	0.35a	17.87e	17.64d	0.23a
	20–40	22.72b	19.3b	3.42a	25.43b	23.06a	2.37b	21.83b	21.69a	0.15a	17.97d	18.29c	−0.33e
	40–60	21.26c	18.76c	2.49c	23.91c	22.25b	1.66c	21.52c	21.39a	0.13a	18.22c	18.41b	−0.18d
	60–80	19.89d	17.49d	2.41c	22.33d	20.55c	1.78c	21.08d	20.66b	0.42a	18.32b	18.44b	−0.12c
	80–100	19.02e	16.97e	2.04d	21.28e	19.76d	1.52c	20.7e	20.34bc	0.35a	18.42a	18.52a	−0.1bc
	100–120	17.88f	16.23f	1.64e	19.93f	18.88e	1.04d	20.12f	19.89c	0.23a	18.34b	18.41b	−0.07b
**2019**	0-20	24.76a	22.52a	2.23a	24.79a	22.00a	2.79a	22.66a	21.61b	1.05a	17.25d	16.31d	0.94a
	20–40	22.51b	20.9b	1.62b	23.58b	21.59b	1.99b	22.26b	21.63ab	0.63b	17.55bc	16.91c	0.65b
	40–60	20.78c	19.99c	0.79c	22.57c	21.53b	1.04c	21.9c	21.74a	0.15c	17.55bc	17.07b	0.48c
	60–80	18.86d	18.15d	0.72cd	21.04d	20.25c	0.8d	20.95d	21.02c	−0.08d	17.58b	17.15b	0.43c
	80–100	17.36e	16.81e	0.56e	19.83e	19.31d	0.52e	20.23e	20.59d	−0.36e	17.51c	17.43a	0.08e
	100–120	16.28f	15.56f	0.71d	18.98f	18.11e	0.87cd	19.82f	19.83e	−0.01d	17.73a	17.39a	0.34d

**Notes.**

Different lowercase letters indicate significant differences at *P* < 0.05 level between different soil layers in each year.

TD represents soil temperature difference between M treatment and NM treatment.

 Two treatments exhibited a relatively large soil temperature difference in the 0–50 cm soil layers during various periods within the two investigated years, and the temperature difference between the treatments gradually decreased with growth progression, displaying a temperature difference of 1.2−1.8 °C in the budding stage, 0.6−1.2 °C in the full blooming stage, and 0.2−0.8 °C in the full bolling stage. The temperature difference in the soil layers below 50 cm was relatively small (between 0.2 and 0.4 °C) in various periods except the budding stage in 2018 with temperature difference of 1.5 °C.

### Temporal and spatial distribution of canopy PAR interception rate

#### Change trend of canopy PAR interception rate over time

**Figure 7:**
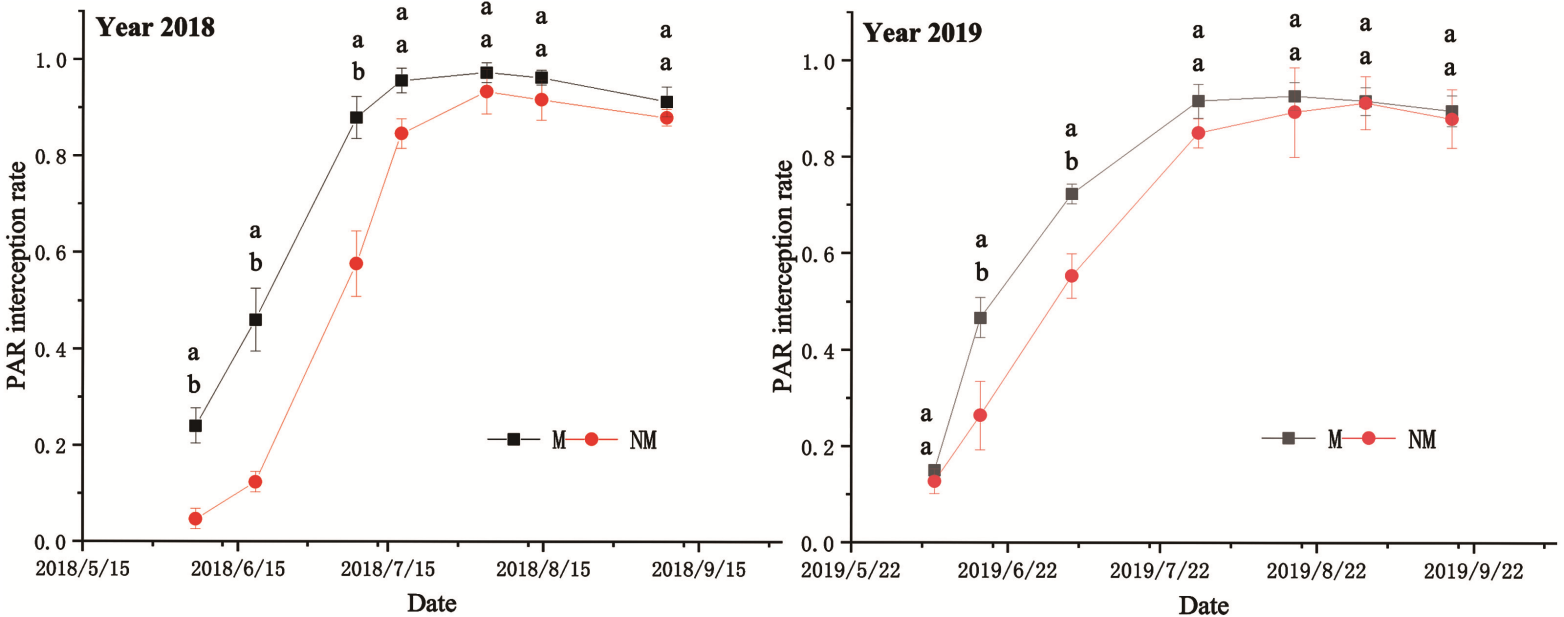
Canopy PAR interception rate in 2018 and 2019. Different lowercase letters indicate significant differences at *P* < 0. 05 level between different treatments.

PAR is the part of solar radiation that can be absorbed by green plants and used for photosynthesis. It is the basic energy source for the formation of biological yield. Therefore, studying the iPAR of different treatments can comprehensively reflect the growth and development of plants and the quality and yield of crop groups. In 2018 and 2019, the canopy iPAR of different treatments showed a downward parabolic shape (Fig. 7). As shown in the figure, the iPAR showed a rapid upward trend before the full bolling stage, and iPAR under the M treatment was significantly higher than that under the NM treatment, followed by a slow decline.

In 2018, the iPAR under the M treatment was significantly higher than that under the NM treatment in the early growth stage. Under the M treatment, iPAR peaked of 0.974 on July 20, which was earlier than that under the NM treatment, while under the NM treatment, it peaked of 0.929 on August 4, which was 4.62% lower than that under the M treatment. After the peak, both treatments exhibited a slow decrease in iPAR. It decreased rapidly under the M treatment but slowly under the NM treatment, resulting in a less obvious difference between the two treatments in the late growth stage. In 2019, the iPAR under M processing increased faster than that under NM treatment before June 20. Afterward, the iPAR under NM treatment was increased rapidly. The difference in iPAR between the two treatments gradually decreased, but the two treatments reached their peaks at different times. The M treatment peaked of 0.926 around July 30, while the NM treatment peaked of 0.909 around September 1, which was 1.8% lower than that of the M treatment. Subsequently, the iPAR under the two treatments exhibited the same downward trend as in 2018. Finally, iPAR under the two treatments was the same.

### Spatial distribution of canopy PAR interception rate

**Figure 8:**
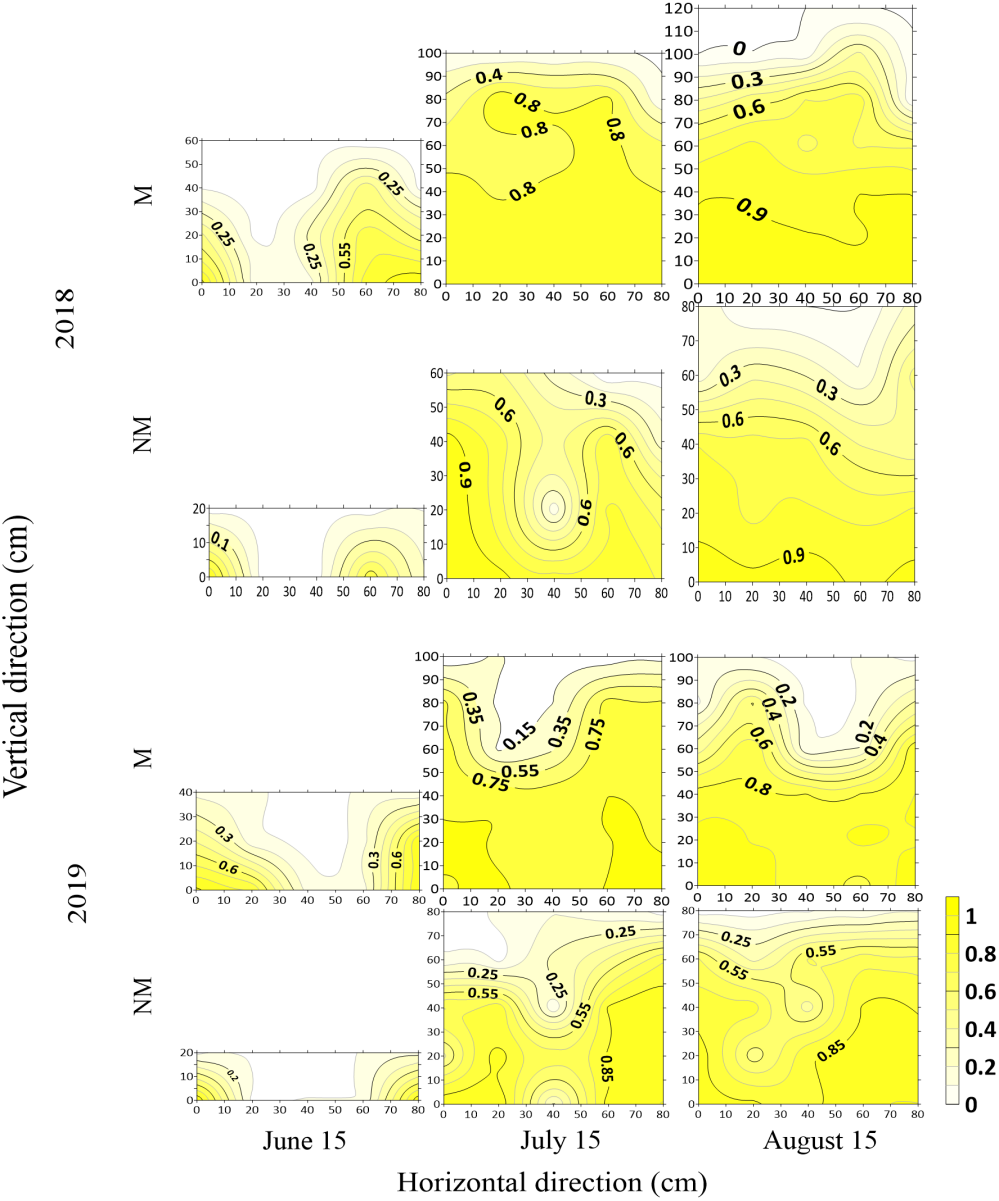
Spatial distribution of canopy PAR interception rate at different growth stages in 2018 and 2019.

The results of different growth stages (June 15, budding stage; July 15 full blooming stage, August 15, full bolling stage) in two years under different treatments showed that with the growth progression of cotton, the spatial distribution of cotton population iPAR changed. Roughly, a “U” shape was observed before the bare ground was shielded in the blooming stage, and light leakage occurred in the middle of the cotton line. With the cotton population increasing, a “V” shape was observed after the bare ground was shielded in the blooming stage. In short, there was a low iPAR in the middle of the cotton rows and a high iPAR close to the cotton rows (as shown in Fig. 8). The iPAR in every growth stage under the M treatment was higher than that under the NM treatment in the same growth period. The iPAR in each growth stage in 2018 was higher than that in the same period in 2019.

Since the iPAR of the cotton population was the accumulation of interception efficiencies at different layers, a higher cumulative PAR interception rate was observed at the bottom layers, and before the bare field was covered by branches, the population interception efficiency increased with the increasing population. Population interception efficiency was close to 1 and remained unchanged after canopy closure. The two-year iPAR distribution diagram showed that the population PAR interception rate in the full bolling stage under the M treatment was close to the maximum (with all higher than 0.9), and the starting position of the PAR interception (group height) was relatively high (approximately 100 cm), while in 2018, under the NM treatment, it also reached more than 0.9 in the full bolling stage, and in 2019, it also reached a maximum of approximately 0.9 in the full bolling stage, but the starting position of PAR interception for the NM treatment was lower than that of the M treatment (at approximately 80 cm), and in the full bolling stage period, it reached approximately 0.85. Moreover, the iPAR of the 40 cm canopy bottom under the M treatment showed little change, whereas, it fluctuated greatly under the NM treatment in the whole canopy.

## Discussion

### Effect of PFM on spatiotemporal changes in soil moisture in cotton fields

The temporal change in soil water content is related to the growth process of cotton. PFM can increase the soil water content by reducing soil water evaporation and redistributing soil moisture (Li et al., 2004; Yan & Li, 2016). In this study, we observed the same trend: the soil moisture in cotton fields under the M treatment was higher than that under the NM treatment. Soil water content was 25% and 13.6% higher under the M treatment than under the NM treatment in 2018 and 2019, respectively. Our results were consistent with the reports by Zong et al. (2021) that PFM increased the water content of topsoil by 23. 83%. Under the M treatment, the soil moisture fluctuated obviously with irrigation and water consumption in cotton fields, which might be because the plastic film prevented the ponding water from entering the soil outside the film (Li, Kang & Yang, 2007), thus promoting full water retention in the soil under film after irrigation. In addition, the cotton population covered by mulching film was larger, and transpiration consumed much water (Xing et al., 2020a; Xing et al., 2020b) thus, there was an obvious fluctuation in the moisture content under the M treatment. However, the soil moisture in the cotton field under the NM treatment was low, and the increase in soil moisture content was not obvious after the same amount of water (as under the M treatment) was supplemented. This might be due to the relatively large evaporation of the soil surface (Zong et al., 2021); thus, during the entire growth period, the moisture content was retained at a low level.

The spatial distribution of soil moisture changed with increasing soil depth, and the entire monitoring profile showed that the fluctuation of soil moisture in the PFM cotton field occurred at deeper soil layers than that in the NM cotton field. The comprehensive test results from the two-year trial indicated that soil moisture in the 0–70 cm deep soil under the M treatment displayed obvious changes, which is consistent with the reports by Li, Kang & Yang (2007) that in the mulched cotton field, the soil water infiltration depth was less than 80 cm for every irrigation. Under the NM treatment, the soil moisture changes mainly occurred in the 0–30 cm soil layer. Due to the function of water retention of PFM (Gao & Li, 2005), the mulched cotton field had higher soil moisture than the nonmulched cotton field in the same growth stage. Therefore, under the condition of supplementing the same amount of water, the film-covered cotton fields had sufficient water to be supplemented into the deep soil for storage. With topsoil water consumption, subsoil water increases over time to replenish, and the soil moisture under the plastic film is consumed and replenished within a larger range, which is in line with the research findings reported by Li et al. (2004) that mulching can redistribute soil moisture. However, since bare soil surface of the nonmulched cotton field, the soil water consumption is consumed by population transpiration and topsoil evaporation (Li, Kang & Yang, 2007). Contrary to the M treatment, the NM treatment due to the deep-layer soil water content was at a lower level, and the upper layer water could not be replenished in time. Therefore, the surface soil water mainly depends on irrigation and precipitation. Only a limited range of the surface soil of the nonmulched cotton field was supplemented with water under the same irrigation amount.

### Effect of PFM on spatiotemporal changes in soil temperature in cotton fields

The temporal change in soil temperature is closely related to the growth period of cotton, and PFM can change the latent heat flux, such as cotton population water evaporation and soil water transpiration (Jiang et al., 2022; Li et al., 2021), and then increase thermal radiation capture and topsoil temperature, especially in the early and middle stages of cotton growth (Zong et al., 2020; Ghosh et al., 2006). In this study, before the initial flowering stage (July 1), especially before the first irrigation (June 20), the temperature of the 0–50 cm soil layer in the cotton field covered with plastic film was higher by approximately 2−4 °C than that in the uncovered cotton field. This result was similar to the reports by Hong (2015) that PFM generally increases the ground temperature by 3−5 °C. Consistently, Zhao et al. (2012) revealed that the daily average surface soil temperature in the covered plots was 2.5−3.2 °C higher than that in the control group in the early growth stage (sowing-seedling). This may be due to the reduction of soil water evaporation and the decrease of latent heat flux by plastic film mulching and then increase the soil temperature (Li et al., 2021). Less obvious effect of PFM on the soil temperature increase (approximately 1−2 °C) of the soil below 50 cm was indicated. With the increasing depth of the entire monitoring soil profile from 0 to 110 cm, the temperature increase effect of the mulching film decreased. This trend is in line with the previous findings by Zhu et al. (2019) and Gao (2021) that there is a certain hysteresis in the response of soil temperature at different depths to air temperature changes and that soil temperature is positively correlated with the accumulated temperature of air temperature in the past, and such a correlation decreases with increasing depth.

Spatial distribution of soil temperature in different soil layers was different with the growth progression, and the temperature difference between different soil layers gradually decreased under the two treatments after the initial flowering period, especially after the full bolling period (August 15). The temperature difference between different soil layers was further reduced to less than 1 °C, which might be for that although there was a certain hysteresis in the response of soil temperature at different depths to air temperature changes (Zhu et al., 2019; Gao, 2021), the deep-layer soil temperature fully increased after the air temperature increased for one month. In addition, the PAR interception rate and the leaf area index (LAI) peaked (Bai et al., 2016) in this period, thus leading to an area decrease in the sun-irradiated mulching film. After the full bolling period, the ground was completely covered by cotton and could not receive direct sunlight (Xia et al., 2021). In addition, some plastic films were broken; thus, the warming and heat preservation effects of film mulching were greatly reduced. After September 12, the surface soil temperature was even lower than the deep layer soil temperature. This result agreed with the finding by Zhao et al. (2012) that in the late growth stage, the difference in soil temperature between different treatments was reduced, and even a negative value occurred. This might be attributed to the hysteresis effect of different correlations between ground temperature at different depths and air temperature (Gao, 2021).

### Effect of PFM on the spatiotemporal changes in the PAR interception rate of the cotton field canopy

The PAR interception rate can reflect the growth and development of plants and the quality of the crop population. PFM is beneficial to the growth of cotton, for example, the associated improvement of leaf area index of cotton and interception rate of PAR reported previously (Xing et al., 2020a; Xing et al., 2020b; Zong et al., 2020). In this study, the population iPAR under the M treatment increased rapidly before the initial flowering stage, especially before June 20, while under the NM treatment, the population iPAR increased slowly during the same period. The possible reason might be the ground temperature was relatively low in the early growth stage (Ghosh et al., 2006), and film mulching could increase ground temperature (Zong et al., 2020), thus benefiting the vegetative growth of cotton and eventually increasing iPAR.

Under NM treatment, with growth progression, the iPAR increased rapidly after June 22, which was higher than that under M treatment, resulting in a gradual decrease in the iPAR difference between the two treatments. Our result is in line with findings by Zhang (1994) that before the first irrigation at the end of June, the cotton root system was basically established at the initial flowering stage, and film-mulched cotton went from vegetative growth into reproductive growth, but the vegetative growth of nonmulched cotton was relatively vigorous at this time.

The iPAR under the two treatments began to decrease after reaching the peak value, however, the inflection point of the two treatments was different, and the decreasing rate was also different. Compared with the NM treatment, the inflection point appeared earlier, and the decreasing rate was faster under the M treatment. This might be mainly due to the warming effect of early mulching (Wang, Kang & Liu, 2003) and the whole-process moisture preservation (Gao & Li, 2005) effect promoting the rapid vegetative growth of cotton, thus entering reproductive growth earlier. However, Li et al. (2016) and Su et al. (2011) have reported that root tended to distribute shallow with film mulching, and the rapid vegetative growth causes excessive consumption of soil nutrients, thus leading to premature aging due to insufficient nutrients and water in the late growth stage, especially during the boll opening period. However, NM treatment promotes roots to go deep into the soil in response to drought stress, resulting in stronger anti-aging ability in the late growth stage.

## Conclusion

The results from this study suggested significant effects of PFM on soil moisture, temperature, and canopy photosynthetically active radiation in cotton fields. The effects of PFM were as follows: (1) PFM could increase soil water content during the whole growth period. The two-year average water content under the M treatment was 13.6% higher than that under NM treatment, and the soil moisture in the topsoil (0–30 cm) changed drastically during the whole growth period. (2) PFM increased the soil temperature by 2−4 °C, especially the topsoil (0–30 cm) temperature in the early cotton growth stages (seedling stage and budding stage). However, the effect of plastic film mulching on temperature was no longer obvious when cotton reached the flowering and Bolling period with the growth progression and the rising air temperature. (3) PFM was beneficial to the growth of cotton, and it improved the PAR interception rate in the budding stage and seedling stage. However, around July 20, the difference in the PAR interception rate between the two treatments gradually decreased, and at the boll opening stage, the PAR interception rate decreased earlier and faster under the M treatment than under the NM treatment.

From the perspectives of cotton production and farmland ecological environment protection, we suggest that in the case of adopting plastic film mulching the irrigation quota and frequency could be appropriately decreased; otherwise, the irrigation quota and frequency should be increased. For nonmulching cultivation mode, management measures should be taken to improve the soil temperature before the middle July. However, the quantification of agronomic management measures under different cropping patterns requires further investigation.

##  Supplemental Information

10.7717/peerj.13894/supp-1 Supplemental Information 1 Raw data

10.7717/peerj.13894/supp-2 Supplemental Information 2 Raw data for Fig. 4 (Soil moisture) and Fig. 6 (soil temperature) All data files are in ‘.dat’ format. Transformed data files in ‘.grd’ format are also included for drawing.

## Additional Information and Declarations

### Data Availability

The following information was supplied regarding data availability: The raw measurements are available in the Supplementary Files.
